# Molecular lesions in alleles of the *Caenorhabditis elegans lin-11* gene

**DOI:** 10.17912/micropub.biology.000589

**Published:** 2022-06-20

**Authors:** Adrie F. Young, Helen F. Schmidt, Meera V. Sundaram

**Affiliations:** 1 Department of Genetics, University of Pennsylvania Perelman School of Medicine

## Abstract

The LIM homeodomain transcription factor LIN-11 is a key regulator of vulva, uterine, and neuron development in *C. elegans.* Multiple alleles of *lin-11* are available, but none had been sequenced. We found that the reference allele, *n389,* is a 15900 bp deletion that also affects two other protein-coding genes, ZC247.1 and ZC247.2. The frequently used *n566* allele is a 288bp deletion located in an intron and affecting the splice acceptor site.

**Figure 1. Molecular lesions in lin-11 alleles n389 and n566:**
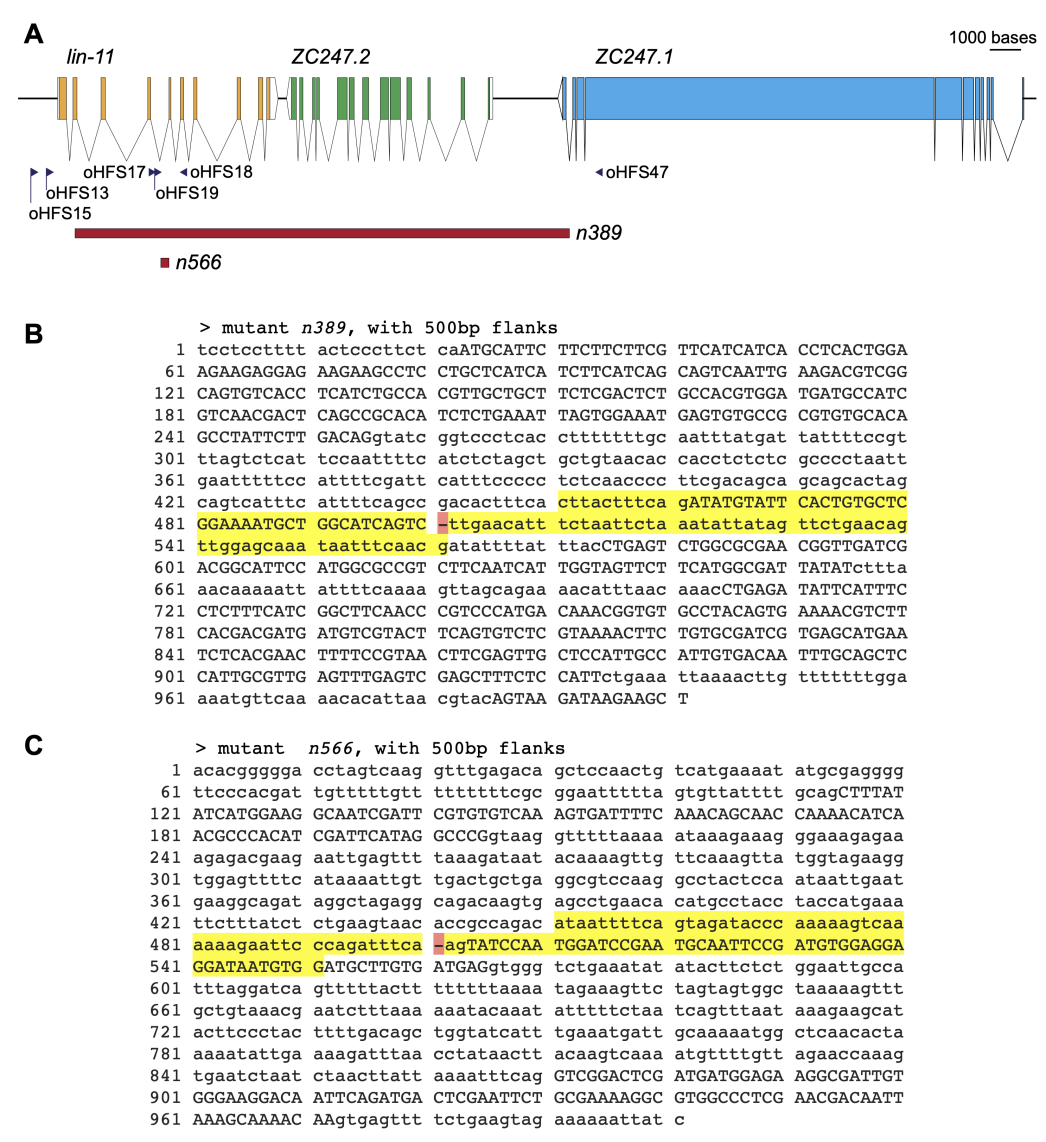
A) Schematic of the right arm of chromosome I in the region of *lin-11* , approximately I:10,247,400-10,281,200. Arrows below indicate the location of primers used in sequencing. Deleted regions of both alleles represented with red boxes. B) Sequence of the *n389* allele with 500 bases displayed on each side of deletion and 50 bases flanking the deletion highlighted in yellow. Red highlight indicates position of deletion. C) Sequence of the *n566* allele, shown as in B. &nbsp;

## Description

LIN-11 is a LIM homeodomain transcription factor (Freyd et al. 1990) expressed in the head muscle, the vulva, the uterus, and some neurons (Freyd, 1991; Hobert et al. 1998; Newman et al. 1999; Sarafi-Reinich et al 2001). Mutations in *lin-11* were first identified by Ferguson and Horvitz (1985) in an EMS mutagenesis screen for defects in vulva development. They identified four alleles of *lin-11* that are 100% egg-laying defective (Egl) as homozygotes: *n382, n389, n566,* and *n672* . Additional *lin-11* alleles *ps1, sy251* and *ty6* also have defects in the egg-laying apparatus (Nelms and Hanna-Rose 2006; Newman et al., 1999). The reference allele is *n389* and considered a null. The *n566* allele is used most often, having 71 appearances in Textpresso compared to 47 for *n389* , possibly because it is the only one of the four original alleles with hermaphrodites that can mate. The additional alleles are not commonly used, with less than five Textpresso appearances each (Textpresso Central (Müller et al. 2018), “ *C. elegans* and Suppl *”* corpus, accessed June 9, 2022). Freyd et al. (1990) had determined by Southern blot that *n389* is lacking the entire probed sequence of ­ *lin-11, n566* is a deletion of about 250bp, *n672* is an insertion of about 5000bp, and that the mutation in *n382* was not detectable by the blot. Despite the common use of *n389* and *n566* , the precise molecular lesions in these alleles had never been reported.

We PCR-amplified and sequenced the *lin-11* genomic region in the *n389* and *n566* alleles. PCR from worm lysates was performed using Herculase Enhanced DNA Polymerase (Agilent, cat#600260). The *n389* allele was amplified using primers oHFS15 and oHFS47 and sequenced using primer oHFS13. The *n566* allele was amplified using primers oHFS17 and oHFS18 and sequenced using primer oHFS19. Sanger sequencing was performed by Penn Genomic Analysis Core DNA Sequencing Facility.

We found that *n389* is a 15900 bp deletion that affects two additional genes. The left breakpoint is in exon 2 of *lin-11* and the right breakpoint is between exons 10 and 11 of ZC247.1 (Fig. 1 A & B). The intervening gene, ZC247.2, is deleted entirely. ZC247.1 is a repeat-rich protein (SMART (Letunic et al., 2021), accessed June 1, 2022) expressed in many neurons, the distal tip cell, and muscle based on transcriptomic data (Kudlow et al., 2012; Li et al., 2020; Taylor et al., 2021). RNAi knockdown of this gene results in germline defects (Green et al., 2011). ZC247.2 is a coiled-coil protein (SMART (Letunic et al., 2021), accessed June 1, 2022) that is expressed in muscle and some neurons (Blazie et al., 2017; Fox et al., 2007; Li et al., 2020; Smith et al., 2010).

We found that *n566* is a 288bp deletion in the fourth intron of *lin-11* , deleting the C of the splice acceptor site CAG (Fig. 1 A & C). The resulting AAG splice acceptor sequence only occurs in 2% of *C. elegans* introns and binds less well to the U2AF splicing protein than CAG or UAG (Hollins et al., 2005). While we have not measured effects of this change on splicing or LIN-11 protein production, the 100% Egl yet mating competent phenotype of this allele indicates a significant but incomplete reduction in function.

We conclude that neither commonly used *lin-11* allele is a simple knockout of *lin-11.* Phenotypes seen only in the *n389* allele should be examined carefully for possible involvement of the other deleted genes. Future studies should examine more than one allele or use a clean deletion made by CRISPR/Cas9.

## Reagents

Primers

**Table:** 

**Primer name**	**Sequence**
oHFS13	TTCGTGGTCGTTCTTCTTCTTC
oHFS15	CAGAATTACAGAGCTTGCGAAG
oHFS17	CAAGTGAGCCTGAACACATGC
oHFS18	TGCTTTAATTGTCGTTCGAGGG
oHFS19	CTCTGAAGTAACACCGCCAGAC
oHFS47	ACTGTAGGCACACCGTTTGT

Strains

**Table:** 

**Strain**	**Genotype**	**Available from**
N2	*Caenorhabditis elegans, wild type*	CGC
MT633	*lin-11 ( n389 )* I; *him-5 ( e1467 )* V.	CGC
BW837	*unc-29 ( e1072 ) lin-11 ( n566 )* I.	CGC
